# Successful Reduction of Acute Puerperal Uterine Inversion with the Use of a Bakri Postpartum Balloon

**DOI:** 10.1155/2015/424891

**Published:** 2015-04-12

**Authors:** Akinori Ida, Koichi Ito, Yoko Kubota, Maiko Nosaka, Hiroshi Kato, Yoshiyuki Tsuji

**Affiliations:** Department of Obstetrics and Gynecology, Kobe Adventist Hospital, 4-1, 8-Chome, Arinodai, Kita-ku, Kobe 651-1321, Japan

## Abstract

Uterine inversion is a state wherein the endometrial surface is inverted. Although this condition may be observed in nonpregnant women, it most commonly develops at the time of delivery. In the present case, a 37-year-old woman without any remarkable history developed acute puerperal uterine inversion after the successful induction of labor. Following the delivery, she complained twice of severe lower abdominal pain; subsequently, hemorrhage was noted at the site of partial detachment of the placenta. These findings led to a diagnosis of placenta accreta, and the patient developed a state of shock. A Bakri postpartum balloon was inserted into the uterine cavity under ultrasonographic guidance and was filled with physiological saline for treatment of this condition. With this procedure, the uterine inversion was completely reduced and the hemorrhage was stopped. Moreover, no reinversion was observed in the postoperative period. These findings suggest that a Bakri postpartum balloon can be used to noninvasively reduce uterine inversion and prevent its recurrence.

## 1. Introduction

Postpartum hemorrhage (PPH) is defined as bleeding that occurs immediately after the placenta is delivered. PPH remains among the top five causes of maternal death in both developed and developing countries.

Uterine inversion is one cause of PPH. Uterine inversion refers to a state wherein the endometrial surface is inverted. In this condition, the uterine fundus is concave or is descending and inverted, and the inner wall of the uterus may occasionally be exposed to the inside of the vagina or to the vulva. Uterine inversion may be observed even in nonpregnant women, in cases where a tumor arising in the uterine cavity is delivered. In general, however, the term “uterine inversion” refers to puerperal uterine inversion that occurs at the time of delivery. Puerperal uterine inversion develops in the third stage of labor because of excessive traction of the umbilical cord or manual detachment of the placenta, and it is reported to occur at a rate of 1 in 2000–8000 pregnancies [1–3]. Puerperal uterine inversion is a rare condition; however, once it develops, massive hemorrhage from the surface of the detached placenta or massive atonic hemorrhage occurs, which may result in hemorrhagic shock. Thus, uterine inversion is a serious condition that could result in maternal death if not treated urgently. Early diagnosis and treatment are essential for the successful management of this condition; therefore, awareness regarding puerperal uterine inversion is important in daily clinical practice.

Here, we describe our experience with a case wherein acute puerperal uterine inversion caused by placenta accreta was successfully reduced noninvasively with the use of a Bakri postpartum balloon (Cook Medical Incorporated, Bloomington, IN, USA).

## 2. Case Presentation

**Figure 1:**
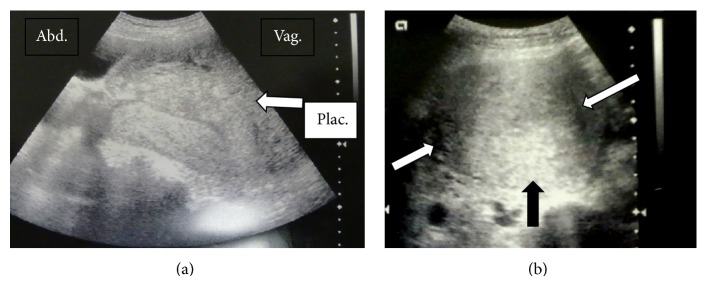
(a) Transabdominal sagittal sonogram showing a mirror image of the uterus with the endometrial pseudostripe represented by the two opposing serosal surfaces [5]. The placenta that adhered to the fundus can be seen on the vaginal side (white arrow). (b) Transabdominal transverse sonogram showing the target sign with the hyperechoic inverted fundus centrally (black arrow) surrounded by hypoechoic fluid (white arrow) between the fundus and vaginal wall [5].

**Figure 2:**
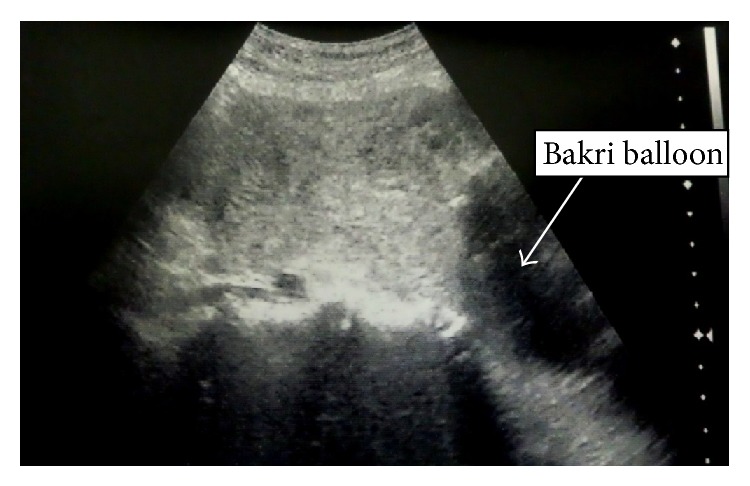
The uterine fundus was pushed up with a balloon containing 300 mL of physiological saline. The shape of the uterine fundus was irregular and indistinct, and complete reduction was not achieved.

**Figure 3:**
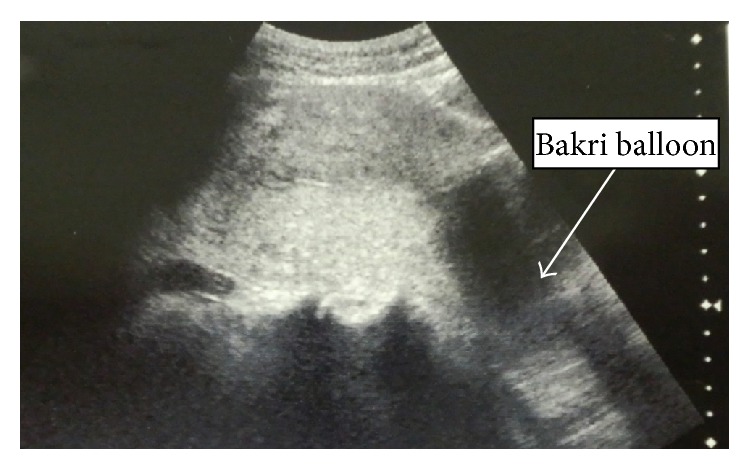
The uterine fundus was again pushed up with a balloon containing 400 mL of physiological saline. The uterus was completely reduced and the hemorrhage was stopped.

The patient was a 37-year-old woman (gravida 1, para 1) without any remarkable medical history. Her last delivery was a normal vaginal delivery, and the expulsion of the placenta was smooth and normal. Her current pregnancy was achieved through frozen embryo transfer. The course of her pregnancy was uneventful. She was admitted to the hospital for rupture of the membranes and induction of labor at 39 weeks and 3 days of gestation. The delivery was completed smoothly, and she gave birth to a baby girl (weight, 3115 g; Apgar score, 9/9) without any problems. Following the World Health Organization's recommendations, we performed controlled cord traction [4]. The placenta was retracted smoothly; however, when approximately half of the placenta had exited through the vagina, she complained of intense lower abdominal pain, and, therefore, the traction was discontinued. Ultrasonography revealed complete uterine inversion, illustrating a “pseudostripe” and a “target sign” as reported by Rana and Patel [5] (Figures 1(a) and 1(b)). She again complained of intense lower abdominal pain. At this time, the fetal membrane appeared dark red and it bulged because of a retroplacental hematoma; thereafter, it ruptured and resulted in a massive hemorrhage. Examination of the placenta that exited the vagina revealed that approximately half of the placenta was still attached to the uterus and that the other half was detached. The hemorrhage originated from the site of detachment. These observations led to a diagnosis of placenta accreta. At this time, the patient developed a state of shock, exhibiting a blood pressure of 68/38 mmHg, pulse rate of 113 beats/min, facial pallor, and shock index of 1.66. The placenta was returned into the vagina, and the patient was transferred to the operating room and was prepared to undergo blood transfusion. Placental removal was attempted by performing mild traction under intravenous anesthesia; however, both the uterus and the placenta descended and exited the vagina, which made the expulsion of the placenta difficult. Therefore, the partially detached placenta was completely removed manually. The endometrium of the uterine fundus was inverted and located outside the vagina, and, thus, complete uterine inversion was noted. Hemorrhage persisted, and the patient's blood pressure decreased; therefore, without using uterine relaxants, reduction was attempted to Johnson's method. The inverted portion was returned to within the uterus and reduced to a state of incomplete inversion, as a better reduction could not be achieved. Although a uterine relaxant was considered to be necessary for reduction in this case, there were concerns about further hemorrhage and hypotension with the use of such relaxants. Therefore, we considered the possibility of achieving hemostasis by using a Bakri postpartum balloon until blood transfusion was accomplished, and achieving reduction by expanding the uterine cavity with the Bakri postpartum balloon. Hence, a Bakri postpartum balloon was inserted into the uterine cavity by holding the base of the balloon with placenta forceps under ultrasonographic guidance. Thereafter, 300 mL of physiological saline was introduced into the balloon, and the placenta forceps holding the balloon were raised toward the uterine fundus; however, complete reduction was not achieved, and hemorrhage persisted (Figure 2). Therefore, 100 mL of physiological saline was additionally injected to increase the total volume of physiological saline to 400 mL, and the placenta forceps holding the balloon were again raised. Consequently, the uterus was completely reduced and the hemorrhage was stopped (Figure 3). Following the uterine reduction, the patient was administered oxytocic agents, and the posttreatment course was carefully observed. No uterine reinversion was observed. Because the placement of the Bakri postpartum balloon was also useful for the prevention of reinversion, the balloon was allowed to remain in the uterus. The vagina was packed with gauze to prevent the expulsion of the Bakri postpartum balloon.

The total hemorrhage volume was 3449 g. The hematologic findings before blood transfusion were as follows: red blood cell count, 128.0 × 10^4^/*μ*L; hemoglobin level, 3.7 g/dL; and hematocrit value, 11.4%. The blood transfusion consisted of 8 units each of red blood cells and fresh frozen plasma.

On the next day, 14 h after the insertion of the Bakri postpartum balloon, the balloon was removed. No reinversion or hemorrhage was observed. The posttreatment course was uneventful.

## 3. Discussion

Puerperal uterine inversion is an extremely rare condition; however, once it occurs, it is very likely to result in massive hemorrhage and a state of shock. Thus, it is a serious and critical obstetric condition that could result in maternal death if early appropriate treatment is not given. Hence, the decision for appropriate treatment needs to be made immediately.

The presentation of puerperal uterine inversion can be acute (within 24 h of delivery), subacute (over 24 h and up to the 30th postpartum day), or chronic (>30 days after delivery) [6]. Puerperal uterine inversion may be classified as four degrees according to the stage of uterine exteriorization [7].

The causes of acute puerperal uterine inversion may be endogenous or exogenous. The endogenous causes include excessive extension of the uterine wall because of placenta accreta, coiling of the umbilical cord, excessively short umbilical cord, multiple pregnancy, exceptionally large fetus, and polyhydramnios; however, these causes are rare. However, most cases of acute puerperal uterine inversion are exogenous, and the condition is often caused by external forces, such as excessive cord traction in the third stage of labor, rough Crede placental expression, and manual detachment of the placenta. Because adhesion between the placenta and the uterus was observed, the uterine inversion in our present case was considered to be caused by placenta accreta.

The representative symptoms of acute puerperal uterine inversion are lower abdominal pain, massive hemorrhage, and shock. These typical symptoms may not be manifested when the degree of inversion is mild; therefore, if hemorrhage of unknown cause persists in the third stage of delivery, the possibility of uterine inversion should be considered.

The diagnosis of acute puerperal uterine inversion is easy if the inner surface of the uterus is exposed to the endocervical canal or the vagina. However, it should be noted that if obstetricians are unaware of uterine inversion, the condition may be misdiagnosed as a submucosal myoma and may thus prevent early diagnosis. The presence or absence of palpability of the uterine fundus immediately after the expulsion of the placenta is useful for the diagnosis. However, if uterine inversion is suspected, ultrasonography should be performed. If “upside down, inside out,” “pseudostripe,” and “target sign,” which indicate that the uterine fundus has dropped into the uterine cavity, are observed, a definitive diagnosis can easily be made (Figures 1(a) and 1(b)).

**Figure 4:**
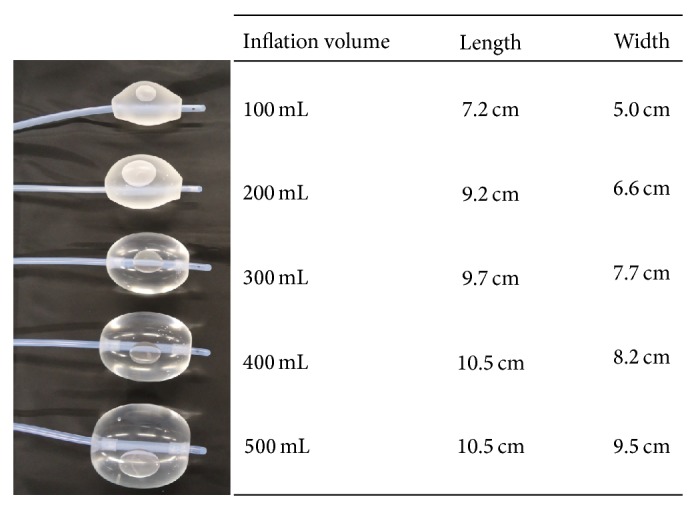
The size of each balloon (inflated to a volume of 100–500 mL).

With regard to the treatment of acute puerperal uterine inversion, methods to rapidly improve systemic shock and simultaneously reduce the inverted uterus should be performed first. Infusion, blood transfusion, antishock therapy, and antidisseminated intravascular coagulation therapy should be performed to improve the systemic conditions. Noninvasive or invasive techniques can be used for the reduction of the inverted uterus. Noninvasive reduction methods, such as the use of manual or hydrostatic pressure, should be attempted first. With regard to manual reduction methods, the method described by Johnson [8] is often used. In addition, a hydrostatic pressure reduction method was previously described by O'Sullivan [9]. Recently, however, Gupta et al. [10] reported a modified hydrostatic reduction method using transurethral resection of the prostate set to reduce failure rate. In contrast, the invasive reduction methods include transvaginal operation according to Spinelli's and Kutner's methods; transabdominal operation according to Huntington's and Haultain's methods; and simple hysterectomy. In the present case, we used a noninvasive method with a Bakri postpartum balloon to successfully reduce the uterus. This method consists of the combination of the principles of Johnson's method and the hydrostatic pressure method. The principle of Johnson's method is as follows: the inverted uterus is raised as much as possible to extend the uterus-supporting ligaments; subsequently, the uterus-supporting ligaments begin to contract, and the inverted uterus is reduced passively by using this contraction force. This reduction method cannot be performed with a lifted fist. The hydrostatic pressure reduction method involves the use of hydrostatic pressure that is generated when physiological saline is infused into the uterus from a higher location. The Bakri postpartum balloon method involves the combination of a hydrostatic pressure effect, in which a balloon filled with physiological saline expands the uterus as a spherical body pressurizing the whole uterine cavity, and the effect of Johnson's method, in which the uterus-supporting ligaments are extended by lifting the balloon as much as possible by using placental forceps. In addition, this method also has a hemostatic effect against atonic hemorrhage, which is the primary role of the Bakri postpartum balloon. Recent studies reported that Bakri postpartum balloon treatment is as effective as uterine artery embolization [11–13]. Uterine inversion may often recur even when the condition is successfully reduced; therefore, the prevention of recurrence is also an important treatment goal. Various methods for preventing reinversion have recently been reported, and the placement of a Bakri postpartum balloon is an effective preventive measure against reinversion [14–17]. The administration of 400 mL of physiological saline, as used in the present case, should produce a spherical body with a diameter of approximately 10.5 × 8.2 cm (Figure 4); therefore, the reduction may be successfully performed through pressurization of the uterus from within the uterine cavity and elevation of the uterus with this spherical body. As the number of female obstetricians has recently increased, the likelihood of these female physicians encountering cases of uterine inversion has also increased. However, a female obstetrician may lack the strength required to raise the inverted uterus with her fist. Therefore, the use of a Bakri postpartum balloon should be extremely helpful in such cases.

Rapid diagnosis and early treatment are the key factors for successfully reducing acute puerperal uterine inversion in patients. Treatment with a Bakri postpartum balloon can be performed promptly with minimal invasiveness and can yield significant hemostatic effectiveness and contribute to a rapid recovery from a state of shock. In the present case, the use of a Bakri postpartum balloon is effective for hemostasis, reduction, and the prevention of reinversion. Moreover, in cases where it is ineffective, it may be easily removed; it may also serve as a temporary option for the patient while preparations for more aggressive treatments are made. Thus, we believe that this procedure involving a Bakri postpartum balloon may be used as a novel noninvasive reduction method for uterine inversion.
